# CogCBR: A Complete Case-Based Reasoning Framework for Wearable-Sensor-Based Gait Screening of Neurodegenerative Diseases

**DOI:** 10.3390/s26134158

**Published:** 2026-07-01

**Authors:** Huayue Liu, Yujia Sun, Lihua Luo, Xingeng Li, Huanghe Zhang

**Affiliations:** 1 The Center for Robotics, School of Control Science and Engineering, Shandong University, Jinan 250100, China; 202200450038@mail.sdu.edu.cn (H.L.); 202300171257@mail.sdu.edu.cn (L.L.); 2 School of Qilu Transportation, Shandong University, Jinan 250100, China; 202300450076@mail.sdu.edu.cn

**Keywords:** case-based reasoning, clinical decision support, wearable sensors, force-sensitive insoles, gait analysis, confidence estimation, case-base maintenance, knowledge containers, edge deployability, Parkinson’s disease, neurodegenerative disease

## Abstract

Wearable force-sensitive insoles enable quantitative gait analysis as a screening aid for neurodegenerative diseases (NDDs), yet prevailing machine learning pipelines give point predictions with no per-case reliability estimate, no intrinsic explanation, and no way to curate their own knowledge base. Case-Based Reasoning (CBR) mirrors clinical reasoning, but deployed healthcare CBR systems typically implement only partial R^4^ cycles, omitting Revise and Retain. We propose CogCBR, a sensor-driven framework that operationalizes the complete R^4^ cycle—Retrieve, Reuse, Revise, Retain—for gait-based NDD screening within Richter’s four knowledge containers, pairing weighted case retrieval with confidence-based clinical triage and a label-verified case-base maintenance policy. On the PhysioNet GaitPDB cohort, CogCBR attains an AUC of 0.861—statistically on par with the strongest tuned baseline under matched tuning, yet the only method evaluated that also provides confidence-based triage, case-based explanation, and longitudinal case-base maintenance, the last validated in a deployment-style streaming simulation. An independent-cohort evaluation on GaitNDD yields an AUC of 0.902; under a stricter cross-modality transfer, however, CogCBR does not exceed the strongest classical baseline, which is also reported. With sub-millisecond inference and a compact footprint, CogCBR suits resource-constrained wearable and edge-health platforms. Prospective longitudinal clinical evaluation and validation in pre-clinical cohorts are left as future work.

## 1. Introduction

Neurodegenerative diseases (NDDs) impose one of the largest and fastest-growing public-health burdens worldwide. Dementia alone affects more than 55 million people globally, and Parkinson’s disease (PD) is estimated to affect approximately 10 million, with prevalence projected to roughly double by 2050 as populations age [1]. Early detection is clinically critical because disease-modifying interventions are most effective when initiated before substantial neuronal loss has occurred [1]. Of particular practical importance is the well-documented fact that motor manifestations, most notably impairments of gait, frequently precede formal diagnosis by several years [2,3,4]. This temporal window between sub-clinical motor change and clinical confirmation defines an attractive opportunity for sensor-based screening support. We emphasise, however, that the cohorts evaluated in this paper consist of patients with established NDD diagnoses (e.g., Hoehn & Yahr stages 2–3) rather than pre-clinical or at-risk subjects, so the present study evaluates gait-based screening support and classification of established NDD-related gait patterns, not pre-symptomatic diagnosis. Demonstrating utility for true early detection would require dedicated pre-clinical cohorts and longitudinal follow-up, which we leave to future work.

Recent advances in low-cost wearable sensing have made quantitative gait analysis a practical screening modality. Force-sensitive insoles instrumented with arrays of piezoresistive transducers and sampled at the centimeter–millisecond scale (typically 100 Hz) produce rich vertical ground-reaction-force (GRF) signals, from which clinically interpretable biomarkers such as stride-to-stride variability, gait-phase timing, inter-limb asymmetry, and stride regularity can be derived [5,6]. Building on such sensor data, machine learning pipelines have progressed from hand-crafted spatial–temporal features classified by linear discriminants [7] and ensemble methods [8], to deep models trained directly on raw GRF time series [9,10]. Although these approaches have steadily improved classification accuracy, they share three limitations that consistently obstruct clinical deployment of sensor-driven screening systems.

**CH1: Lack of intrinsic, case-grounded explanations.** Clinicians require not only a prediction but a rationale that can be verified against domain expertise [11]. Most sensor-based gait classifiers expose only a scalar decision and cannot be inspected as patient evidence; post hoc methods such as SHAP [12] and LIME [13] approximate an opaque model rather than ground decisions in prior patients with similar gait profiles, leaving clinicians without a transparent footing to endorse or override a prediction.

**CH2: Absence of per-prediction reliability assessment.** Existing sensor-based screening models report only aggregate metrics (accuracy, AUC, F1) and offer no mechanism for indicating *when an individual prediction should not be trusted*. In a screening setting, where the costs of a missed early diagnosis and an unnecessary specialist referral are asymmetric, there is no principled way to flag ambiguous cases for expert review or to restrict automated decisions to confident predictions.

**CH3: No mechanism for sustained case-base curation as new sensor data accumulate.** Sensor-driven clinical pipelines are inherently longitudinal: as new patient recordings arrive, the underlying knowledge should be refined accordingly. Current pipelines either retrain monolithically (incurring substantial cost and risking concept-drift instability) or accept new data uncritically (introducing label noise and self-confirmation bias). Neither strategy provides a principled way to identify low-quality recordings and either rescue them through expert relabeling or retire them.

Case-Based Reasoning (CBR) [14,15] offers a natural framework that addresses CH1–CH3 simultaneously. The R^4^ cycle, namely Retrieve, Reuse, Revise, and Retain, closely mirrors clinical reasoning: physicians recall similar prior patients, adapt previous assessments, evaluate confidence in their conclusion, and update experiential knowledge as new patients are seen [16,17]. Crucially, every CBR prediction carries an *intrinsic* explanation in the form of the retrieved cases and the features driving similarity, addressing CH1 directly [18,19]; the *Revise* stage formally evaluates prediction reliability and routes uncertain cases for expert review, addressing CH2; and the *Retain* stage curates the case base over time under an explicit quality model, addressing CH3. In practice, however, most deployed healthcare CBR systems have stopped at *Retrieve* and *Reuse* [20,21,22]; that is, CBR has typically been used as a similarity-based classifier rather than as a complete reasoning cycle. The Revise and Retain containers, which are precisely the components that would close CH2 and CH3, have remained on the methodological shelf in clinical practice.

A complementary view of completeness is given by the four *knowledge containers* of Richter [23]: *vocabulary* (features describing cases), *similarity* (how cases are compared), *case base* (the stored experience), and *adaptation* (how stored solutions are transferred). Because these containers are interchangeable, a well-curated case base reduces the need for elaborate adaptation knowledge. In clinical screening, where explicit adaptation rules are difficult to elicit, this trade-off motivates investing in similarity (Retrieve) and case-base curation (Retain) while keeping adaptation lightweight—a perspective we adopt explicitly in CogCBR.

**Figure 1:**
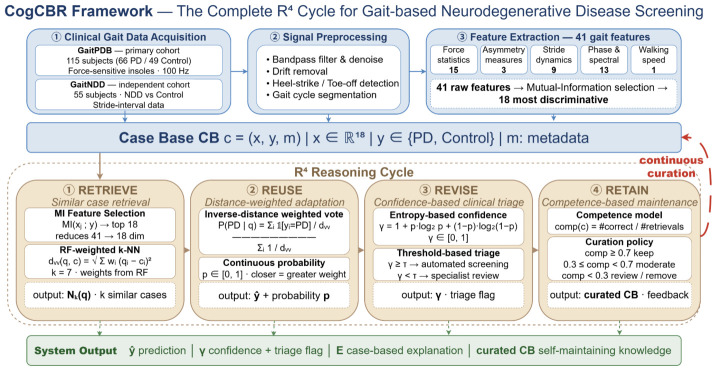
Overview of the proposed sensor-driven CBR framework CogCBR. Raw vertical ground-reaction-force signals from bilateral force-sensitive insoles (100 Hz) are pre-processed and converted into a 41-dimensional feature vector that is reduced to the 18 most discriminative features via mutual-information (MI) selection, forming the case base. The R^4^ reasoning cycle then proceeds: **Retrieve** (RF-weighted *k*-NN over the MI-selected features), **Reuse** (inverse-distance weighted voting yielding a continuous PD probability), **Revise** (entropy-based confidence and threshold-based triage to specialist review), and **Retain** (competence-based curation that updates the case base via the dashed feedback arrow). The system outputs a screening prediction, a triage flag, a case-based explanation, and a curated case base.

To address CH1–CH3, we propose CogCBR (Cognitive CBR), a sensor-driven CBR framework that implements the complete R^4^ cycle for gait-based NDD screening from wearable force-sensitive insoles. CogCBR is framed throughout by the four knowledge containers and instantiates each stage of the R^4^ cycle with components well established in the CBR literature: mutual-information (MI) feature selection together with Random Forest (RF)-weighted *k*-NN retrieval (Retrieve, addressing CH1 by anchoring decisions in retrieved prior patients), inverse-distance weighted voting that yields a continuous probability (Reuse), entropy-based confidence estimation that flags low-certainty predictions for specialist review (Revise, addressing CH2), and competence-based maintenance under an explicit augmentation policy that admits only label-verified cases (Retain, addressing CH3). The novelty lies not in any individual operator but in their principled integration into a single sensor-driven pipeline, closed by a triage and augmentation policy that links confident predictions, expert review, and case storage. Figure 1 illustrates the complete CogCBR pipeline.

The main contributions of this paper are summarized as follows:

- We propose a complete, clinically grounded R^4^ CBR cycle for wearable-sensor-based NDD screening support. To the best of our knowledge, CogCBR is the first system in this domain to operationalize the *Revise* and *Retain* containers jointly with Retrieve and Reuse, closing the loop between automated screening, expert review, and longitudinal case-base curation.
- We design an entropy-based confidence-driven triage mechanism that supports a coverage-vs.-accuracy trade-off appropriate for clinical workflows, and pair it with a conservative case-base augmentation policy that admits only label-verified cases (confident self-predictions confirmed at clinical follow-up, or low-confidence cases adjudicated by a specialist), preventing label noise and self-confirmation bias. At τ=0.1, CogCBR retains 68.7% of predictions with accuracy improving from 80.0% to 87.3% on PhysioNet GaitPDB. We validate the longitudinal behavior of this policy in a deployment-style streaming simulation (Section 4.8), in which the label-verified rule avoids the label-noise accumulation of naive self-training and matches an all-true-label no-curation reference while bounding case-base growth.
- We conduct quantitative comparison against 16 baselines, comprising 14 generic ML/DL methods and two re-implemented domain-specific gait-classification pipelines [7,8], under stratified 10-fold cross-validation on PhysioNet GaitPDB and an independent-cohort reproducibility evaluation on GaitNDD. CogCBR attains AUC = 0.861 on GaitPDB and AUC = 0.902 on GaitNDD, achieving competitive performance on both datasets—statistically on par with the strongest tuned baselines on GaitPDB under a matched-tuning comparison (Section 4.3). Under a stricter cross-modality source-to-target transfer (train on GaitPDB, test on GaitNDD), CogCBR does not exceed the strongest classical baseline; this source-to-target result is reported in Section 4.9.
- We profile the computational cost and deployment footprint of CogCBR against representative baselines. With 0.080 ms per-sample inference latency and an 8.4 KB deployment payload, CogCBR is well-suited to wearable-sensor and edge-health scenarios in which storage, latency, and interpretability are simultaneously constrained.
- We deliver intrinsic, case-based explanations grounded in clinically interpretable sensor-derived features. Each prediction is accompanied by the retrieved similar patients, the gait features driving similarity, and the direction of clinical deviation.

The remainder of this paper is organized as follows. Section 2 reviews related work on CBR for clinical decision support, CBR for movement disorders, and sensor-based gait screening for PD. Section 3 describes the materials and methods, including the GaitPDB and GaitNDD datasets, the sensor acquisition and signal-processing pipeline, the four knowledge containers of CogCBR, the experimental protocol, and the baselines. Section 4 reports the experimental results on GaitPDB and GaitNDD, the Revise-stage triage analysis, the computational-cost profiling, and the system-component ablation. Section 5 discusses the implications of the findings, statistical caveats, and the limitations of the present cross-sectional study. Finally, Section 6 concludes the paper and outlines directions for future work.

## 2. Related Work

### 2.1. CBR for Clinical Decision Support and the Four Knowledge Containers

CBR has a long history of application in medical and health-science domains [16,17,21,24]. The R^4^ cycle [14,20,25] parallels clinical decision-making: physicians recall prior patients, adapt previous assessments, verify outcomes, and update their experiential knowledge accordingly. As Watson [25] argued, CBR is fundamentally a *methodology* for problem solving rather than merely a classification technique.

The four knowledge containers of Richter [23] provide a complementary view of CBR system design. Because the containers are interchangeable, strengthening the case base reduces the need for complex adaptation, and inverse-distance weighted reuse over an appropriate similarity measure becomes sufficient [26]. This trade-off directly informs CogCBR’s design: we invest in vocabulary (MI feature selection), similarity (RF-weighted distance), and case-base (competence-based maintenance) while keeping adaptation lightweight (inverse-distance voting). The motivation is pragmatic: explicit adaptation rules are difficult to elicit in clinical settings, whereas vocabulary, similarity, and case-base quality can each be operationalized from sensor data.

CBR explanations are *intrinsic*: the retrieved cases themselves constitute the explanation, and the similarity features provide a clinically grounded vocabulary [18,19,27]. This is structurally distinct from feature-attribution methods such as SHAP [12] and LIME [13], which approximate the behavior of an opaque model *post hoc*: CogCBR’s explanations are exemplar-based explanations rooted in the actual reasoning process, in the tradition of CBR explanation work [18,19,27].

Our exemplar-based explanations can also be situated within the broader landscape of explainable medical AI. Prototype-learning networks such as ProtoPNet [28] and deep prototype models for case-based reasoning [29] learn a set of representative prototypes and classify a query by its similarity to them, echoing the case-based intuition but requiring end-to-end representation learning; nearest-neighbor and exemplar-retrieval techniques for neural-network explainability [27] instead attach example-based justifications to an already-trained opaque model. A recurring argument across this literature is that, for high-stakes clinical decisions, inherently interpretable models are preferable to post hoc explanations of black-box predictors [30]. CogCBR sits at the inherently-interpretable end of this spectrum: its exemplars are actual prior patients rather than learned latent prototypes, so each explanation is faithful to the retrieval-and-voting process by construction and requires no separately trained explanation model.

### 2.2. CBR for Parkinson’s Disease and Movement Disorders

Several prior systems have applied CBR to clinical diagnosis and, more recently, to PD. Begum et al. [21] surveyed CBR in the health sciences and noted that, although CBR is well-suited to clinical reasoning, most deployed systems implement only Retrieve and Reuse. Marling and Whitehouse [31] applied CBR to care planning for Alzheimer’s disease patients, demonstrating case retrieval over patient records. Saraiva et al. [22] proposed a hybrid CBR/rule-based reasoning system for the early diagnosis of gastrointestinal cancer, but did not include systematic confidence assessment or case-base maintenance. A common pattern across these systems is a focus on retrieval and reuse with limited attention to revision (confidence assessment) and retention (competence-based curation), precisely the gap that CogCBR addresses.

Beyond classification accuracy, the CBR literature has produced principled techniques for the Revise and Retain containers that have rarely been operationalized in deployed clinical systems. Smyth and McKenna [32] formalized competence models for case-base maintenance; Smyth and Keane [33] introduced competence-preserving forgetting; Leake and Wilson [34] categorized maintenance dimensions; and Delany et al. [35] studied case-base editing for noise reduction. CogCBR draws on this body of work to instantiate Retain in a clinical screening pipeline driven by wearable-sensor data.

### 2.3. Sensor-Based Gait Screening for Parkinson’s Disease

Quantitative gait analysis has emerged as a promising sensor-based biomarker for NDDs. Hausdorff et al. [5,36] demonstrated that stride-to-stride variability measures derived from foot-worn force sensors distinguish NDD patients from healthy controls. Subsequent work has explored temporal, spectral, and nonlinear-dynamics features derived from force-sensitive insoles and inertial measurement units [3,6]. Machine learning approaches have progressed from spatial–temporal feature classifiers [7] and ensemble methods [8] to deep learning on the raw sensor time series [9,10]. These models typically optimize classification accuracy alone, leaving the question of *when to trust a prediction* unaddressed and providing no mechanism for curating the case base as new sensor recordings accumulate. To enable a direct quantitative comparison rather than the merely descriptive comparison common in this literature, we re-implement the published pipelines of Wahid et al. [7] and Rehman et al. [8] as baselines (Section 3.9), in addition to 14 generic ML/DL methods. More broadly, methodological experience from adjacent wearable- and physiological-sensing domains is directly relevant to the design choices made here. In smart-insole-based human-activity recognition, D’Arco et al. [37] show that careful feature selection over combined plantar-pressure and inertial channels improves classification robustness while reducing dimensionality on exactly the sensor modality used here; and in multimodal physiological-signal analysis, Iranfar et al. [38] combine feature selection, outlier detection, imputation, and classification to cope with the missing-data and sensor-variability problems that also arise in wearable gait recordings. These works reinforce the feature-engineering, feature-selection, and quality-control choices in CogCBR’s vocabulary and case-base containers.

## 3. Materials and Methods

CogCBR implements the complete CBR cycle [14] for gait-based NDD screening. We describe the design through Richter’s four knowledge containers: *vocabulary* (Section 3.1 and Section 3.2), *similarity* (Section 3.3), *adaptation* (Section 3.4), and the *case base* together with its quality maintenance (Section 3.6). Section 3.9 then details the experimental protocol used to evaluate the framework.

### 3.1. Clinical Case Representation (Vocabulary Container)

#### Sensor Data and Datasets

We use the **Gait in Parkinson’s Disease (GaitPDB)** database from PhysioNet [6] as the primary dataset, and the **Gait in Neurodegenerative Disease (GaitNDD)** database, also from PhysioNet, as an independent validation cohort. GaitPDB comprises 66 PD patients (Hoehn & Yahr stages 2–3) and 49 age-matched healthy controls (115 subjects in total). Each subject walked at a self-selected pace on level ground while wearing bilateral force-sensitive insoles; each insole contains eight piezoresistive pressure transducers that record the vertical ground reaction force at 100 Hz. The PhysioNet records also include per-foot total forces (in newtons). We primarily use the left-foot total force, the right-foot total force, and their sum (the bilateral total force) as the basis for gait-event detection and feature extraction, because these channels most stably reflect limb loading, heel strike, and toe-off events during walking. Each subject contributes a single feature vector to the case base, so cross-validation splits are at the subject level by construction.

### 3.2. Sensor Acquisition and Signal-Processing Pipeline

**Figure 2:**
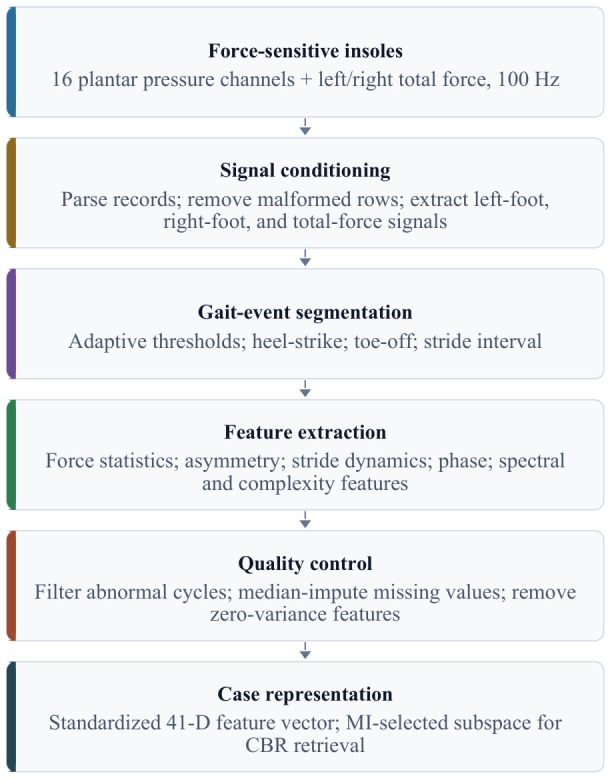
Sensor-to-feature processing pipeline. Raw vertical ground-reaction-force signals from bilateral force-sensitive insoles (16 plantar-pressure channels plus per-foot total force at 100 Hz) are conditioned, segmented into gait events by adaptive thresholding, and converted into mechanical, temporal, phase, spectral, and complexity features. After missing-value imputation, abnormal-cycle filtering, and standardization, each subject is represented by a clinically interpretable 41-dimensional gait case vector, which is then reduced to 18 dimensions by mutual-information selection for downstream CBR retrieval.

Figure 2 summarizes the sensor-to-feature pipeline that converts raw force-sensitive-insole signals into the case representation used by CogCBR. The pipeline comprises five stages: signal conditioning, gait-event segmentation, feature extraction, quality control, and case representation.

**Signal conditioning.** Each raw recording is parsed line by line. Rows with anomalous formatting or insufficient columns are discarded, and recordings with no valid samples or with too few samples to support stride-level analysis are excluded. Let $F_{L}\left(t\right)$ and $F_{R}\left(t\right)$ denote the total vertical GRF under the left and right foot, respectively, and let $F_{T}\left(t\right)=F_{L}\left(t\right)+F_{R}\left(t\right)$ denote the bilateral total force. A bandpass filter is applied to suppress high-frequency sensor noise and low-frequency baseline drift before further processing.

**Gait-event segmentation.** To detect gait events robustly across subjects with different body weights and walking styles, we adopt an adaptive amplitude threshold derived from each subject’s own signals. A foot is considered to be in contact with the ground when its total force exceeds 20% of its mean force, i.e., $F_{L}\left(t\right)>0.2\;{\bar{F}}_{L}$ and $F_{R}\left(t\right)>0.2\;{\bar{F}}_{R}$. Rising edges of the resulting binary contact signal are taken as candidate heel-strike events and falling edges as candidate toe-off events. The stride interval for each foot is computed as the time between consecutive heel-strikes of the same foot. To suppress spurious events caused by noise or partial foot lifts, only physiologically plausible stride intervals between 0.4 s and 3.0 s are retained. The double-support phase is estimated as the proportion of samples in which both feet are in contact simultaneously.

**Feature extraction.** From the segmented signals, we extract 41 features organized in five clinically interpretable categories:

1. **Force statistics** (15 features): mean, standard deviation, coefficient of variation (CV), skewness, and kurtosis of the left-foot, right-foot, and total force signals, characterizing plantar load magnitude and its variability.
2. **Asymmetry measures** (3 features): force asymmetry index, left–right force ratio, and inter-foot phase synchronization, capturing the gait asymmetry commonly observed in PD.
3. **Stride dynamics** (9 features): mean stride interval, stride variability (CV), cadence, stride asymmetry, left and right stride CV, autocorrelation at lag 1, and separate left/right stride regularity measures, reflecting rhythmic stability of walking.
4. **Gait phase and spectral features** (13 features): stance percentage, swing percentage, double-support percentage, swing-to-stance ratio, dominant frequency, spectral entropy, harmonic ratio, stride regularity, step regularity, sample entropy, and stride irregularity, describing phase structure, periodicity, and signal complexity.
5. **Walking speed** (1 feature): self-selected gait velocity from the clinical metadata.

Each feature has a known physiological interpretation and an established association with NDD pathology [3,4], and each is directly derivable from the wearable-sensor signal stream.

**Quality control.** Missing feature values caused by short recordings, anomalous segments, or failed event detection are imputed with the per-feature median. Features that are entirely missing or have zero variance after imputation are removed. Because subsequent CBR retrieval depends on distance computation, all retained features are standardized (z-score) before similarity calculation, so that scale differences do not dominate the distance.

**Case representation.** After this pipeline, the raw force-sensor stream of each subject is reduced to a low-dimensional, clinically interpretable case vector spanning the loading, asymmetry, stride-variability, rhythm, double-support, and signal-complexity changes that characterize NDD gait. Compared with raw high-dimensional time-series input, this representation preserves clinical meaning while remaining well-matched to small-sample CBR retrieval, explanation, and confidence estimation.

#### Case Structure

Each case c=(x,y,m) comprises a feature vector $x\in R^{d}$ (with d=18 after MI selection), a class label y∈{PD,Control}, and optional clinical metadata *m*. The metadata field *m* carries *contextual* information that is excluded from the similarity computation but preserved for clinical interpretation; concretely, *m* may include age, sex, height, weight, body-mass index, recording site, recording date, walking-trial protocol (overground/treadmill/dual-task), and, where available, disease subtype and Hoehn & Yahr stage. The metadata is displayed alongside retrieved neighbors in the explanation output (Section 3.7) so that clinicians can flag demographic mismatches between the query and its neighbors, but is excluded from the distance computation to prevent retrieval being biased by demographic confounders.

### 3.3. Retrieve (Similarity Container)

The Retrieve stage identifies relevant prior cases via two phases that operate in different knowledge containers: vocabulary refinement (MI feature selection) and similarity weighting (RF-weighted distance).

#### 3.3.1. Mutual Information Feature Selection

We apply mutual information (MI) [39] as a univariate filter to select the most discriminative gait features. For each feature $x_{j}$ we compute $\mathrm{MI}(x_{j};y)$ and retain the top *n* features by MI score (n=18, determined by grid search over n∈[5,41]; see Section 4.11). This reduces the feature space from 41 to 18 dimensions, mitigating the curse of dimensionality that is particularly acute in small-sample CBR [40]. For the primary experiments, we report a fixed-parameter protocol used throughout the main comparison; the optimism that may arise from selecting the MI feature count on the evaluation folds is acknowledged as a methodological caveat in Section 5.

#### 3.3.2. RF-Weighted *k*-NN Retrieval

Given a query case *q*, we retrieve the *k* nearest neighbors [41,42] from the case base CB using a weighted Euclidean distance:

(1) $$d_{w}\left(q,c\right)=\sqrt{\sum_{i=1}^{d}w_{i}{\left(q_{i}-c_{i}\right)}^{2}}$$

where $w_{i}$ is the importance weight of feature *i*, derived from a Random Forest (RF) classifier [43] (100 trees) trained on the case base.

#### 3.3.3. Justification for Random Forest Weights

Wettschereck et al. [44] review feature-weighting methods for instance-based learners along three axes: (i) wrapper vs. filter, (ii) global vs. local, and (iii) supervised vs. unsupervised, and observe that supervised global wrappers tend to outperform filter-only weights when features differ in scale and relevance. Among supervised options (information-theoretic weights, conditional entropy, gain ratio, RELIEF, and ensemble-based importances), we adopt RF-derived importance for three reasons. (1) It is a supervised, global, *multivariate* estimator that captures feature interactions, complementing the univariate MI filter. (2) It is robust to small samples and to the mixed monotonic/non-monotonic feature–label relationships present in gait data (e.g., walking speed is strongly monotonic with the label, while harmonic ratio interacts with stride dynamics). (3) It is stable under bootstrap resampling, important for the 10-fold protocol in which weights are recomputed per fold. We additionally compare against alternatives (Cosine, Manhattan, plain Euclidean, NCA) in Section 4.11, where RF-weighted Euclidean is the strongest empirically.

### 3.4. Reuse (Adaptation Container: Distance-Weighted Voting)

Adaptation in CogCBR is intentionally lightweight: a strong vocabulary, similarity measure, and case base together reduce the need for elaborate adaptation knowledge [23,26]. The Reuse stage adapts the solutions of the retrieved cases via inverse-distance weighted (IDW) voting, in which closer neighbors exert greater influence:

(2) $$P\left(\mathrm{PD}|q\right)=\frac{\sum_{c_{i}\in N_{k}\left(q\right)}\frac{⊮[y_{i}=\mathrm{PD}]}{d_{w}\left(q,c_{i}\right)+\epsilon }}{\sum_{c_{i}\in N_{k}\left(q\right)}\frac{1}{d_{w}\left(q,c_{i}\right)+\epsilon }}$$

where $N_{k}\left(q\right)$ denotes the *k* nearest neighbors of *q* and $\epsilon =10^{-8}$ prevents division by zero. IDW voting yields the continuous probability estimates required by the entropy-based confidence in the Revise stage (Section 3.5).

### 3.5. Revise (Confidence-Based Clinical Triage)

CogCBR quantifies prediction reliability through an entropy-based confidence score. For predicted probability p=P(PD∣q):

(3) $$\mathrm{confidence}\left(q\right)=1+p\log_{2}p+\left(1-p\right)\log_{2}\left(1-p\right)$$

Confidence ranges from 0 (maximum uncertainty, at p=0.5) to 1 (complete certainty). Cases falling below a configurable threshold τ are flagged for specialist review rather than receiving an automated classification, so that confident predictions support screening decisions while uncertain cases are routed to a human expert. We emphasize that τ is a deployment-time parameter; the values reported in Section 4.4 sweep τ for descriptive purposes and should not be read as the recommended operating point of a deployed system, which would require independent calibration on a target cohort.

### 3.6. Retain (Case-Base Container Under an Augmentation Policy)

The Retain stage maintains case-base quality [33,34,35]. Following Smyth and McKenna [32], we compute a competence score for each case *c*:

(4) $$\mathrm{competence}\left(c\right)=\frac{\left|\{q:c\in N_{k}\left(q\right)\wedge \hat{y}\left(q\right)=y\left(q\right)\}\right|}{\left|\{q:c\in N_{k}\left(q\right)\}\right|}$$

which is the fraction of correct predictions among all queries for which *c* was retrieved as a neighbor. Cases with high competence (≥0.7) are confirmed as valuable; low-competence cases (<0.3) become candidates for review or removal, as they may correspond to noisy recordings or borderline subjects. These thresholds (0.7/0.3) define the *operational maintenance policy* of the Retain stage; in Section 4.7, we additionally report finer descriptive bins (0.8/0.5) to summarize the static competence distribution, but those bins do not constitute a separate Retain policy.

**Case-base augmentation policy.** A key design question is when, and under what conditions, the case base is augmented during the cycle. CogCBR adopts a deliberately conservative policy:

- *Outside individual reasoning cycles*, periodic competence maintenance is run over the current case base. Cases with competence <0.3 are flagged for clinical review rather than silently removed, and the final removal/retention decision is approved by a specialist. This separation, with maintenance batched outside the per-query cycle, follows the architectural distinction emphasized by Leake and Wilson [34].
- *Confident predictions* (γ≥τ) are **not** added to the case base on their own. Without ground truth they could only reinforce existing biases (a self-training pathology). Such queries enter the case base only after their label has been confirmed at clinical follow-up.
- *Low-confidence predictions* (γ<τ) are routed to a specialist via the Revise stage. The specialist’s adjudication supplies the ground-truth label, after which the case is added to the case base. This is the primary mechanism by which the case base grows in deployment, since Revise actively surfaces precisely those cases that the current case base does not yet cover well.

This policy aligns Retain with the knowledge-container view: the case base is updated only with cases whose label is reliable, and Revise’s triage decides which queries are worth eliciting expert labels for. The dashed feedback arrow in Figure 1 corresponds exactly to this update path. We validate the longitudinal effect of this policy in a deployment-style streaming simulation (Section 4.8) that goes beyond the cross-sectional evaluation reported here; prospective clinical validation remains future work.

### 3.7. Clinical Explanation Output

Following the goals of explanation in CBR [18,19], CogCBR generates a structured explanation containing (i) the *k* most similar cases together with their labels and distances; (ii) the top three features contributing most to each similarity, identified by $w_{i}\left|q_{i}-c_{i}\right|$; and (iii) the direction of deviation (higher/lower). A representative explanation reads: “Patient X has gait patterns similar to previous PD cases Y and Z, with reduced walking speed and increased stride variability.” The explanation is thus grounded in clinically interpretable sensor-derived features and in actual prior patients, rather than in a learned attribution model.

### 3.8. Algorithmic Summary

Algorithm 1 summarizes the complete CogCBR pipeline.
**Algorithm 1** CogCBR Pipeline.**Require:** 
Query *q*, case base CB, MI feature set F, RF weights w, neighborhood size *k*, confidence threshold τ**Ensure:** 
Prediction $\hat{y}$, confidence γ, explanation *E*, augmentation flag *a*  1:**Retrieve.** Select MI features: $q^{\prime }\leftarrow q\left[F\right]$  2:Compute $d_{w}\left(q^{\prime },c^{\prime }\right)$ for all c∈CB {Equation (1)}  3:$N_{k}\leftarrow$*k* cases with smallest $d_{w}$  4:**Reuse.** p← weighted vote over $N_{k}$ {Equation (2)}  5:$\hat{y}\leftarrow ⊮\left[p\geq 0.5\right]$ {binary class prediction}  6:**Revise.** $\gamma \leftarrow 1+p\log_{2}p+\left(1-p\right)\log_{2}\left(1-p\right)$ {Equation (3)}  7:**if** γ<τ **then**  8:    Flag for clinical review; a← await_specialist_label  9:**else**10:    a← await_followup_confirmation11:**end if**12:**Explain.** E←∅13:**for** $c_{i}\in N_{k}$ **do**14:    top3← 3 features with largest $w_{j}\left|q_{j}^{\prime }-c_{i,j}^{\prime }\right|$15:    $E\leftarrow E\cup \{\left(c_{i},y_{i},d_{w},\mathrm{top}3\right)\}$16:**end for**17:**Retain** (offline batch). Periodically recompute Equation (4); flag low-competence cases for specialist review; admit query *q* into CB only once its label has been confirmed (per *a*).18:**return** $\hat{y}$, γ, *E*, *a*

### 3.9. Experimental Protocol

#### 3.9.1. Datasets

**GaitPDB** [6] comprises 115 subjects (66 PD, 49 controls) with ground-reaction force recorded by bilateral force-sensitive insoles at 100 Hz, as described in Section 3.1; the classification task is binary PD vs. Control. **GaitNDD** comprises 55 subjects (20 Huntington’s disease, 16 controls, 13 ALS, 6 PD) with stride-interval data from PhysioNet and is used here as an *independent-cohort reproducibility cohort* (within-cohort 10-fold CV; see Section 4.9 for the distinction from a strict source-to-target transfer experiment). The classification task on GaitNDD is binary *any-NDD vs. Control* (39 vs. 16 subjects), consistent with the screening framing of GaitPDB; multi-class subtype classification (PD/HD/ALS) is not attempted because the per-subtype sample sizes (notably $n_{\mathrm{PD}}=6$) are too small to support reliable subtype-specific evaluation.

#### 3.9.2. Cross-Validation Protocol

We employ stratified 10-fold cross-validation with a fixed random seed (42) for reproducibility. Each subject contributes a single feature vector and appears in exactly one fold, so within-subject leakage is impossible by construction. On each fold, the training partition (∼103 subjects on GaitPDB) serves as the CogCBR case base, and the RF feature weights are recomputed on the training partition only.

For the primary experiments, we report the fixed-parameter protocol used throughout the main comparison. The optimism that may arise from selecting the MI feature count and neighborhood size on the evaluation folds is acknowledged as a methodological caveat in Section 5; we additionally quantify it directly in a nested cross-validation audit (Section 4.3), which recomputes median imputation, standardization, MI ranking, RF feature-weight estimation, and the selection of *n* and *k* strictly inside each training fold.

#### 3.9.3. Baselines

We compare CogCBR against three groups of baselines.

*Group A: 10 traditional ML methods.* Logistic Regression, SVM (RBF and Linear kernels), Random Forest, XGBoost [45], GBM, Decision Tree, *k*-NN (k=5), MLP, and Naive Bayes (default scikit-learn [46] hyperparameters; features standardized). We deliberately use defaults to limit researcher degrees of freedom on the baseline side; the implication is a tuning asymmetry against CogCBR (whose *k*, *n*, and distance metric are tuned, Section 4.11), which we discuss in Section 5.

*Group B: 4 deep learning baselines.* Deep MLP (128–64–32, BatchNorm, Dropout, 200 epochs), 1D-CNN (32/64 filters, kernel size 3), LSTM (2-layer, hidden size 64), and TabNet [47] ($n_{a}=n_{d}=16$, 100 epochs). All deep models are trained with the Adam optimizer and class-weighted loss on an NVIDIA RTX 4090 GPU. Together with Group A (10 methods) and Group C (2 methods), this gives the 16 baselines referenced throughout the paper.

*Note on the LSTM baseline.* Because the engineered features are atemporal, an LSTM cannot exploit its sequential inductive bias on them directly. The configuration reported as “LSTM” in Table 1 therefore treats the 41 feature dimensions as a length-41 input sequence with one channel, providing the most direct comparison with the other tabular baselines. As a sensitivity analysis, we additionally trained an “LSTM-raw” variant on the original force time series (down-sampled to 1 Hz, padded to 600 time steps, per-foot vertical GRF as channels) using the same architecture. LSTM-raw reaches AUC = 0.781±0.072 on GaitPDB, statistically indistinguishable from the tabular LSTM (Wilcoxon p=0.39), suggesting that the small-sample regime is the dominant bottleneck for deep sequence models here. LSTM-raw is reported in Table 1 as a supplementary analysis and is *not* counted among the 16 baselines used for the multiple-comparison correction.

*Group C: 2 domain-specific gait baselines.* We re-implement two published gait-classification pipelines as direct comparison points: *Wahid-2015* [7] (spatial–temporal feature engineering followed by linear/quadratic discriminant classification) and *Rehman-2019* [8] (clinically curated gait features with a tuned Random Forest classifier and recursive feature elimination). Both are evaluated under the identical 10-fold protocol. Re-implementations follow the algorithmic descriptions in the original papers; small differences from the original numbers are expected because the underlying datasets and pre-processing pipelines differ.

*GaitNDD evaluation.* For GaitNDD, the input is stride-interval data only; baselines that require multi-channel force input or that failed to converge on n=55 are reported as “—” in Table 1. Specifically, SVM (Linear), MLP, Deep MLP, 1D-CNN, LSTM, LSTM-raw, and TabNet either failed to converge stably on GaitNDD or required input modalities not available there, and are therefore omitted rather than reported with degenerate scores.

#### 3.9.4. Metrics and Statistical Tests

We report AUC (primary), accuracy, F1-score, sensitivity (recall), and specificity. Sensitivity is particularly relevant in a clinical screening setting because it directly reflects the false-negative (missed-diagnosis) burden. Statistical significance is assessed via the Wilcoxon signed-rank test (one-sided, $H_{1}$: CogCBR > baseline) on per-fold AUC scores. We report uncorrected per-baseline *p*-values throughout. With 16 baselines on GaitPDB, the Bonferroni-corrected significance level is α/16≈0.003; in Section 5, we identify which comparisons survive this correction so that readers can calibrate the strength of the per-baseline claims. Because the comparisons are directional and exploratory on small datasets, the *p*-values are used only to contextualize the AUC ranking rather than as conclusive significance tests.

## 4. Results

### 4.1. Gait Feature Differences Between Groups

**Figure 3:**
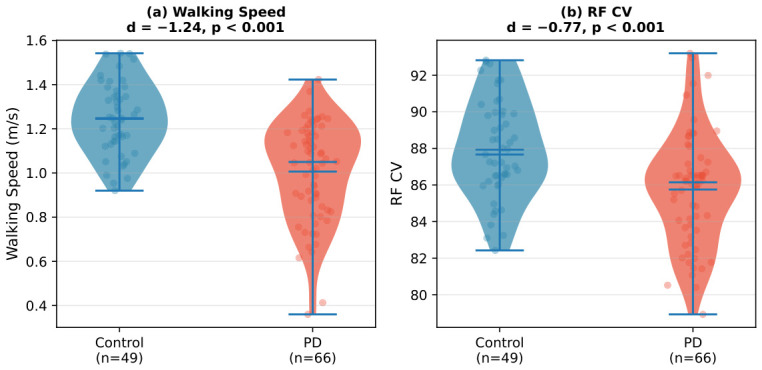
Distribution of two discriminative gait features. (**a**) Walking speed; (**b**) right-foot force CV. Violin plots show the full probability density; individual data points are overlaid with jitter and boxplots indicate quartiles. PD patients exhibit significantly lower walking speed and altered right-foot force-variability (lower RF CV in PD relative to controls in this cohort) than controls.

Figure 3 illustrates the distribution of two discriminative gait features between PD patients and healthy controls. Walking speed shows a large effect size (Cohen’s d=−1.24), with PD patients exhibiting significantly reduced velocity. Right-foot force CV (RF CV) also differs substantially (d=−0.77): PD patients show systematically lower RF CV values than controls in our cohort, indicating altered force-variability patterns rather than uniformly higher variability. Both differences are statistically significant (p<0.001). The direction of the RF CV effect should not be over-interpreted: gait variability in PD is a multi-dimensional construct, and stride-timing variability (captured separately by stride-CV features) often increases in PD even when plantar-force CV does not. In plain terms, a reader might expect Parkinsonian gait to be *more* variable on every measure, so a lower right-foot force-CV in patients can seem counter-intuitive. The two quantities measure different things: force-CV captures cycle-to-cycle variation in how hard the foot presses, whereas stride-CV captures variation in step timing. Many PD patients walk with smaller, more uniform (shuffling) plantar-force profiles, which lowers force-CV, while their step timing becomes more irregular, which raises stride-CV. A decrease in one therefore does not contradict the increased gait variability classically associated with PD.

### 4.2. Screening Performance

**Figure 4:**
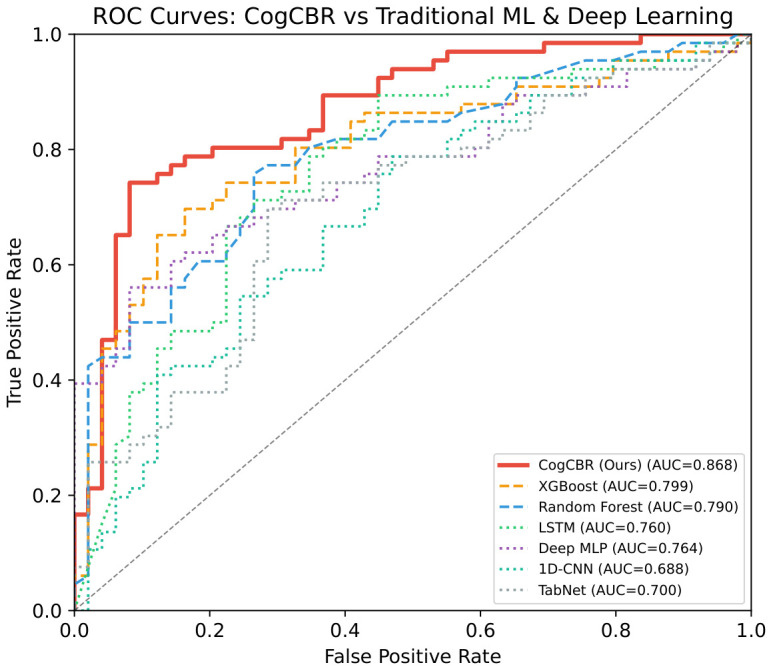
ROC curves comparing CogCBR with the top traditional ML and deep learning baselines on GaitPDB. AUC values shown in this figure are computed on predictions pooled across folds and therefore differ marginally from the per-fold means reported in Table 1 (e.g., 0.868 pooled vs. 0.861 per-fold mean for CogCBR).

Table 1 presents classification performance on GaitPDB and GaitNDD. CogCBR attains AUC = **0.861** on GaitPDB (mean of per-fold AUCs) (The value 0.868 in Figure 4 is computed on predictions *pooled across folds* rather than as the mean of per-fold AUCs. We report the per-fold mean (0.861) as the primary number because it pairs naturally with the per-fold Wilcoxon test, and the pooled value (0.868) where a single ROC curve or threshold sweep is presented) and achieves competitive top-ranked performance among the 16 baselines. Per-baseline pairwise Wilcoxon tests yield uncorrected p<0.05 against 11 of 14 generic baselines and against both re-implemented gait-specific baselines (*Wahid-2015*: 0.798, p=0.031; *Rehman-2019*: 0.821, p=0.049). These per-baseline *p*-values are uncorrected; both the multiple-comparison correction and the tuning asymmetry between CogCBR (whose *k*, *n*, and distance metric are tuned) and the Group A defaults are discussed in Section 5. The intended reading is that CogCBR is *competitively ranked* and additionally provides functionality, including confidence-based triage (Revise), case-base maintenance (Retain), and intrinsic case-based explanations, that the baselines do not.

On GaitNDD, CogCBR attains AUC = 0.902 and achieves the highest mean AUC among the eleven methods that can be evaluated on stride-interval data; however, its margin over the strongest classical baselines (Random Forest 0.887, Rehman-2019 0.872) is not statistically significant. This absence of separation is addressed explicitly in Section 5: on a 55-subject dataset the per-fold AUC variance (±0.111) is comparable to the absolute AUC differences (∼0.015–0.030) the test would have to detect.

For the screening-relevant sensitivity/specificity trade-off, CogCBR achieves sensitivity = 0.745 and specificity = 0.880 on GaitPDB, and sensitivity = 0.897 and specificity = 0.688 on GaitNDD. These complementary profiles illustrate that the model is conservative on the larger PD-vs-control cohort (favoring few false positives) and more sensitive on the smaller NDD-vs-control cohort (favoring few false negatives), consistent with the screening framing in which uncertain cases are routed to specialist review by the Revise stage rather than acted on as final diagnoses.

Figure 4 shows the ROC curves on GaitPDB.

**Figure 5:**
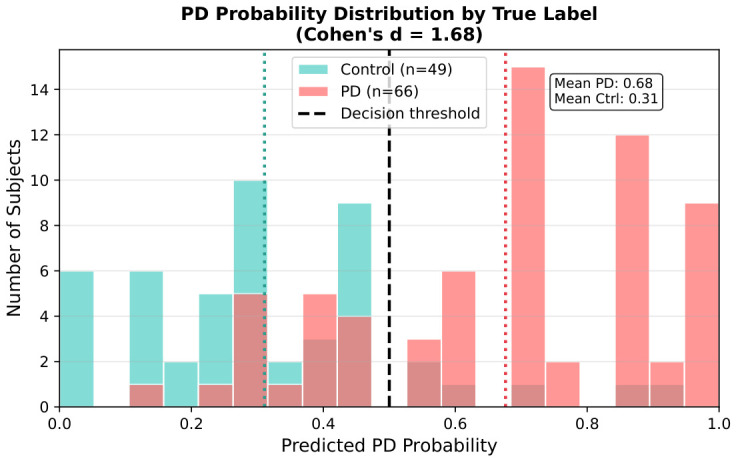
Distribution of predicted PD probabilities by true label. The two distributions are well-separated (Cohen’s d=1.68), and most predictions fall clearly on the correct side of the 0.5 decision threshold.

Figure 5 shows the distribution of predicted PD probabilities for each class. The two distributions are well-separated (Cohen’s d=1.68), with PD patients centered around 0.68 and controls around 0.31.

### 4.3. Parameter-Selection Optimism and Baseline Fairness

This section examines three methodological issues: selection optimism from choosing *n* and *k* on the evaluation folds, the fairness of comparing a tuned CogCBR against default-configured baselines, and the strength of the multiple-comparison evidence. Each is evaluated through a nested cross-validation audit, a fully tuned-vs.-tuned comparison, and a corrected-vs.-uncorrected significance summary.

To quantify the optimism introduced by selecting the MI feature count *n* and the neighborhood size *k* on the evaluation folds, we repeated the full pipeline under a strictly nested protocol [48,49]: on each outer training fold an inner 5-fold loop selected *n* and *k*, and median imputation, standardization, MI ranking, and RF feature-weight estimation were all recomputed inside the training partition only. Table 2 reports the result. On GaitPDB, the nested AUC is 0.842±0.093, only 0.019 below the fixed-parameter value of 0.861, indicating that the selection optimism is small; the nested estimate remains at or above the strongest classical baselines evaluated under their default configuration (for example, GBM 0.836 and Rehman-2019 0.821). On GaitNDD, the nested AUC is 0.835±0.168; the larger drop and wide interval are consistent with the 55-subject cohort. We retain the fixed-parameter numbers as the primary results, with this audit confirming they are not materially inflated by selection.

To assess baseline fairness, a fully tuned-vs.-tuned comparison (Table 3 and Table 4) was conducted: each Group A baseline was given the same inner-CV tuning budget that CogCBR receives. Under matched tuning, CogCBR’s nested AUC (0.842) remains the highest on GaitPDB, but its margin over the strongest tuned baseline (GBM, 0.836) is not significant (one-sided Wilcoxon p=0.242). On the smaller GaitNDD cohort, tuned Random Forest (0.913) ranks highest and CogCBR (0.835) ranks fifth; however, the per-fold AUC variance is large (SD up to ±0.24 on 55 subjects), so the ranking is not statistically separable and no comparison survives Bonferroni correction. CogCBR is therefore competitive with—and statistically indistinguishable from—the strongest tuned classical baselines, with its contribution resting on the integrated Revise, Retain, and explanation functionality that the higher-AUC baselines do not provide.

Finally, to make the multiple-comparison evidence explicit (Table 5), we summarize, for each of the 16 baselines on GaitPDB, the uncorrected one-sided Wilcoxon *p*-value and whether it remains significant at the uncorrected level (α=0.05) and at the Bonferroni-corrected level (α/16≈0.003). Thirteen of the sixteen comparisons are significant before correction, but only the 1D-CNN comparison (p=0.002) survives Bonferroni correction. We therefore present the uncorrected pattern as suggestive rather than confirmatory, consistent with the claims stated in Section 5.

### 4.4. Confidence-Based Clinical Triage (Revise)

Table 6 reports the confidence/coverage/accuracy trade-off produced by the Revise stage. At threshold ≥0.1, CogCBR retains 68.7% of predictions with accuracy improving from 80.0% to 87.3% and (pooled) AUC from 0.868 to 0.907. At ≥0.6, the 13.9% most-confident predictions correspond to only ∼16 subjects pooled across folds, on which the system happens to be perfectly accurate; the AUC of 1.000 at this threshold should therefore be read as a property of the easiest-to-classify subset of the cohort rather than as a calibrated estimate of operator behavior. Clinicians can adjust the threshold to balance throughput against safety, but the threshold itself must be calibrated on data independent of any future deployment cohort: an external calibration set is required before any specific value of τ can be recommended for clinical use.

**Figure 6:**
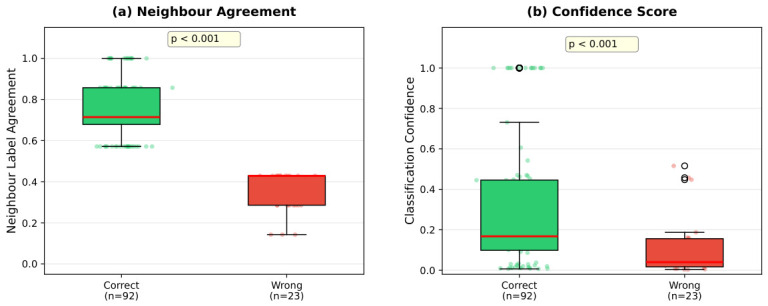
Revise mechanism validation. (**a**) Neighbor label agreement; (**b**) classification confidence, both compared for correctly classified vs. misclassified cases. Both metrics are significantly higher for correct predictions (p<0.001), enabling identification of uncertain cases for specialist referral.

Figure 6 further validates the Revise mechanism by comparing correctly classified with misclassified cases. Neighbor label agreement (the proportion of *k* neighbors sharing the query label) shows substantial separation, with 0.75±0.14 for correct predictions versus 0.34±0.10 for misclassifications (Mann–Whitney p<0.001). Classification confidence is 2.7× higher for correct cases (0.32 vs. 0.12).

### 4.5. Calibration of the Confidence Score

**Figure 7:**
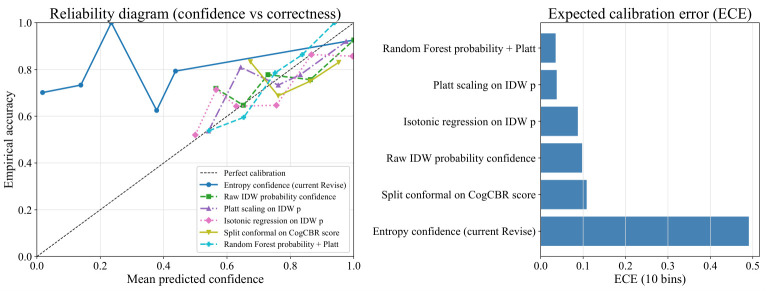
Calibration of the CogCBR confidence score on GaitPDB. (**Left**) Reliability diagram (mean predicted confidence vs. empirical accuracy); the diagonal is perfect calibration. (**Right**) Expected calibration error (ECE, 10 bins; lower is better). The entropy confidence (current Revise) is deliberately a triage/separation score rather than a calibrated probability, which is why its ECE bar is large; post hoc calibration of the IDW probability (Platt, isotonic, RF-probability + Platt) and split conformal prediction on the CogCBR score yield well-calibrated or validly covered estimates when a calibrated risk is required.

We do not claim that the entropy-based confidence score is well-calibrated in the strict probabilistic sense, and a calibration analysis (Table 7, Figure 7) confirms this. Read as a probability of correctness, the entropy confidence is systematically under-confident (ECE =0.491): it collapses toward zero near p=0.5 even though such predictions remain frequently correct, reflecting its role as a triage/separation score (Section 3.5, Figure 6) rather than a calibrated probability. The same analysis shows that, when a calibrated probability is required, post hoc calibration of the IDW probability achieves low calibration error (RF-probability + Platt: ECE =0.035; Platt: ECE =0.038) and split conformal prediction attains valid, slightly conservative coverage (93.3% at a 90% target), at a modest accuracy cost on this small cohort. We therefore retain the entropy confidence for triage, where separation rather than calibration is what matters, and recommend post hoc calibration for any deployment that must report calibrated risk estimates. The calibrators compared are Platt scaling [50], isotonic regression [51], and split conformal prediction [52], assessed by the expected calibration error [53] and the Brier score [54].

### 4.6. Clinical Explanation Through Case Studies

We present three representative cases from the 10-fold CV evaluation, chosen to illustrate the structure of the explanation output and the safety value of Revise.

*Case A: correct PD detection (confidence = 1.0).* Subject #4 (PD) is correctly classified with maximum confidence. All seven retrieved neighbors are PD cases (nearest distance = 0.97). The explanation highlights low walking speed (0.68 m/s), reduced stride regularity (0.68), and elevated double-support time (38.4%), all established PD gait biomarkers [3]. The most discriminative features are mean total force (weight 0.048), stride regularity (0.065), and total-force CV (0.033).

*Case B: correct control classification (confidence = 1.0).* Subject #81 (Control) is correctly classified, with all seven neighbors being controls (nearest distance = 0.53), distinguished by normal walking speed (1.35 m/s), high stride regularity (0.70), and low double-support time (22.7%).

*Case C: Revise preventing a misclassification.* Subject #109 (Control) is misclassified as PD (P(PD)=0.90), but Revise assigns only moderate confidence (0.53). Under a threshold of ≥0.6, this case would be flagged for specialist review rather than acted on. Inspection reveals that six of the seven neighbors are PD patients, despite normal walking speed (1.37 m/s) but anomalous stride regularity (0.10) and zero double-support time, suggesting possible data-quality issues with the sensor recording. Revise thus prevents a false positive that could trigger an unnecessary referral.

### 4.7. Case Competence Analysis (Retain)

The Retain stage evaluates each case’s classification utility across all 10-fold CV queries. Under the operational policy thresholds defined in Section 3.6 (≥0.7 keep, 0.3–0.7 monitor, <0.3 flag for specialist review), the 115 cases distribute as summarized in Table 8. Cases that are never retrieved as a neighbor in the 10-fold evaluation are assigned competence = 0 by convention and so fall in the lowest bin; this convention means low-competence cases comprise both genuinely error-prone retrievals and cases that the current case base has rendered redundant. In either case, the augmentation policy’s specialist-review pathway provides the same audit mechanism. We note in passing that the 68.7% (79/115) of cases in this confirmed-retain group coincides numerically with the 68.7% Revise-stage coverage at τ=0.1 (Table 6); although numerically identical, the two figures are computed independently from different quantities—case competence (Equation (4)) here versus entropy confidence (Equation (3)) there—so their coincidence carries no special meaning.

For completeness, we additionally report a finer descriptive grouping that is used only to summarize the static distribution of case-base quality (and is *not* a separate maintenance policy): 62 cases (53.9%) have competence ≥0.8 (clearly high quality); 43 cases (37.4%) lie in 0.5–0.8 (moderate); and 10 cases (8.7%) lie below 0.5 (lower quality, meriting closer inspection). The mean competence is 0.768 (SD = 0.265). The cases with competence <0.3 are a strict subset of the 10 cases below 0.5; lower-quality cases plausibly represent atypical gait patterns, borderline subjects, or sensor-recording artifacts.

This analysis is *descriptive*: it characterizes the static case base under the 10-fold protocol and identifies which cases would be candidates for review. It does not, on its own, validate the longitudinal effect of the augmentation policy, which would require a deployment-style simulation in which the case base grows under realistic clinical-feedback rates. This simulation is provided in Section 4.8.

### 4.8. Longitudinal Retain Simulation

To validate the augmentation policy beyond the static analysis of Section 4.7, we simulate deployment as a stream [55]: from a seed case base, GaitPDB subjects arrive sequentially (10 random orderings), CogCBR predicts and triages each, and the case base is updated under the policy of Section 3.6. We compare the policy (A) against naive self-training that admits confident predictions with their predicted labels (B), a verification ablation following A’s data flow but with unverified confident labels (B2), an all-true-label reference that performs no curation (C), and a static no-growth baseline (D), sweeping the follow-up confirmation rate (the clinical-feedback rate) over {0.3,0.5,0.7,1.0} at τ=0.1 (with τ=0.2 as a sensitivity check). As summarized in Table 9, the label-verified policy A injects zero label noise versus ≈11 erroneous labels for B, beats B on the area under the learning curve across all feedback rates (paired *p* down to 0.002), and clearly exceeds the static baseline D (0.801–0.838 versus 0.750), and approaches the all-true-label no-curation reference C in final AUC—matching it within the per-fold noise at confirmation rates ≥0.7 and marginally exceeding it at full confirmation, since A (unlike C) additionally curates the case base—while maintaining a smaller case base (60.8–80.9 versus 86 cases) and showing the same qualitative pattern at τ=0.2. The verification ablation B2 degrades as more unverified labels enter (2.5 to 9.7 erroneous labels); the gap to A is negligible at low feedback rates but widens in A’s favor as the rate rises (A improving while B2 degrades), isolating label verification as the operative mechanism. This remains a resampling-based simulation on a cross-sectional cohort; prospective longitudinal clinical validation is still required.

**Figure 8:**
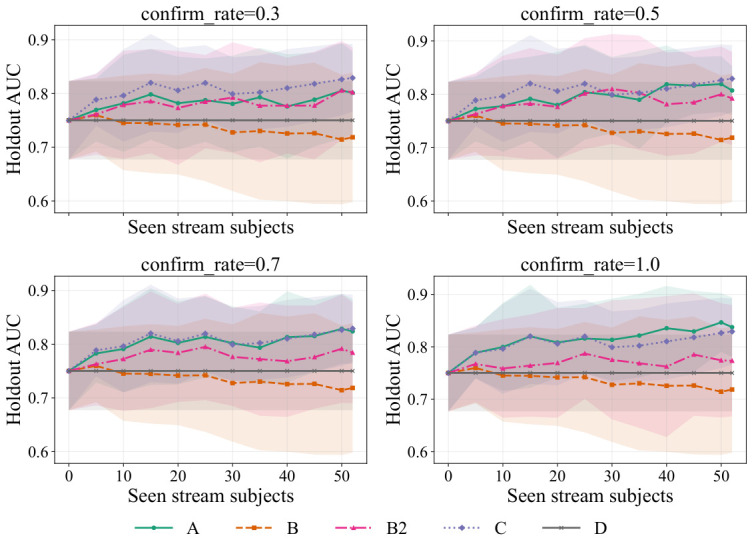
Held-out AUC versus number of arrived subjects under the Retain streaming simulation (τ=0.1; mean over 10 random stream orderings, shaded bands denote ± one standard deviation), for each follow-up confirmation rate: label-verified policy (A), naive self-training (B), verification ablation (B2), all-true-label no-curation reference (C), and static no-growth baseline (D).

Figure 8 shows the corresponding held-out AUC as a function of the number of arrived subjects: across all four feedback rates, the label-verified policy A rises above the static baseline D toward the all-true-label no-curation reference C, while naive self-training B falls below D and the verification ablation B2 degrades as the feedback rate increases.

### 4.9. Independent-Cohort Reproducibility Evaluation on GaitNDD

We further evaluated CogCBR on GaitNDD, a second independent PhysioNet dataset. This experiment uses stratified 10-fold cross-validation *within* GaitNDD; following the recommendation that the term “external validation” should be reserved for source-to-target transfer with non-overlapping cohorts and matching label definitions, we describe this experiment as an *independent-cohort reproducibility evaluation*. It assesses whether the same CogCBR workflow can be reproduced on an independent cohort with stride-interval features rather than as a strict external test. The GaitNDD feature set is small (nine stride-interval features); MI selection retained eight of them, dropping a single near-zero-variance feature. With k=3, CogCBR achieves AUC = 0.902±0.111 (Table 1), with the highest mean AUC among the eleven evaluable methods. The triage and explanation mechanisms transfer unchanged. Statistical significance against the strongest baselines (Random Forest 0.887, Rehman-2019 0.872) is not reached, and the per-fold variance is comparable to the absolute AUC differences. The honest reading is that CogCBR is competitive but not measurably superior on GaitNDD under within-cohort CV; we revisit this in Section 5.

#### 4.9.1. Choice of *k* on GaitNDD

For GaitNDD, we used k=3 rather than the k=7 setting employed on GaitPDB. The motivation is structural rather than tuning-driven: GaitNDD is substantially smaller (55 subjects) and provides only a nine-feature stride-interval representation. In each training fold, the minority control class contains approximately 14–15 subjects; using k=7 would therefore make each neighborhood cover almost half of the available control class and risk over-smoothing local retrieval evidence. A smaller, more local neighborhood (k=3) preserves the case-based explanation property under the reduced feature space and cohort size. We acknowledge this dataset-specific choice as a limitation in Section 5 and note that future larger-cohort studies should select *k* under a fully nested protocol.

#### 4.9.2. Source-to-Target Transfer

As a stricter cross-modality domain-shift analysis, we trained models on GaitPDB and tested them directly on GaitNDD using only the shared stride-based features. In this setting, CogCBR obtained AUC = 0.737, *below* the strongest baseline (Logistic Regression, AUC = 0.832); detailed results are reported in Table 10. This confirms that source-to-target transfer across different sensor modalities and label definitions is substantially harder than within-dataset validation, and that CogCBR does not exceed classical baselines in this regime. Similarity-based retrieval inherits the limitations of its case base when the deployment distribution differs sharply from it; our main claims therefore rest on GaitPDB cross-validation and the within-cohort reproducibility evaluation on GaitNDD, not on direct cross-modality transfer. We report the transfer numbers transparently rather than omit them.

### 4.10. Computational Cost and Edge Deployability

Beyond predictive performance and interpretability, practical clinical screening systems must be deployable under resource-constrained settings such as wearable sensors, mobile health terminals, and bedside screening devices. We therefore profile CogCBR and representative baselines in terms of training time, single-sample inference latency, and deployment payload size on the GaitPDB analysis set (115 subjects). Inference latency is averaged over 2000 single-sample runs, matching the per-subject deployment scenario rather than batch throughput. For CogCBR, the deployment payload comprises the case base with 18 MI-selected features, class labels, standardization parameters, selected feature indices, and RF-derived feature weights.

Table 11 reports the results. CogCBR has the lowest single-sample inference latency in the comparison (0.080 ms): more than 30× faster than Random Forest (2.678 ms) and at least 4× faster than XGBoost (0.510 ms), 1D-CNN (0.426 ms), and LSTM (0.369 ms). It also has the smallest deployment payload at 8.4 KB; Random Forest, 1D-CNN, and LSTM require 48.1 KB, 35.0 KB, and 211.7 KB, respectively. Although XGBoost is also compact at 15.0 KB, it provides neither case-level explanations, neighbor retrieval evidence, nor confidence estimation. Training cost is comparable to that of traditional ML baselines (all non-deep methods fit within 0.1 s); we do not interpret small fitting-time differences as a primary result, since they fall within wall-clock measurement variability.

CogCBR’s case base grows linearly with the number of cases; on the present GaitPDB cohort the full deployment payload is under 10 KB and a single inference takes under 0.1 ms. Even if the case base were extended to several thousand subjects, storage would remain manageable, and the Retain stage bounds case-base growth through competence-based curation. CogCBR is therefore well positioned as a lightweight, interpretable, and continuously maintainable edge screening method for rapid first-line risk assessment of neurodegenerative disease.

### 4.11. System Component Analysis

We systematically evaluate each CogCBR component. The values of *n* and *k* reported below were selected at their AUC peaks on the same 10-fold partitions, a protocol asymmetry already declared in Section 3.9 and acknowledged in the limitations (Section 5).

#### 4.11.1. MI Feature Selection

MI ranking identifies walking speed as the most discriminative feature (MI = 0.150), followed by force CV (0.091), stride regularity (0.089), and double-support percentage (0.088), all clinically established PD gait markers [3]. Grid search over n∈[5,41] finds that AUC peaks at n=18 (0.861) and declines with more features (n=41: AUC = 0.801), confirming that low-MI features inject noise into the distance computation in small-sample CBR.

#### 4.11.2. Distance Metric

Table 12 compares five distance metrics for case retrieval (MI-18, k=7). The RF-weighted Euclidean distance outperforms all alternatives (+0.048 AUC over the second-best Cosine), confirming that supervised, multivariate feature importance in the distance function improves retrieval. NCA, a learned metric, performs worst, consistent with overfitting on the small training set.

#### 4.11.3. Neighborhood Size

AUC peaks at k=7 (0.861) and declines both for smaller values (k=3: AUC = 0.795 with higher variance) and for larger values (k=15: AUC = 0.838 with over-smoothing).

#### 4.11.4. Component Ablation

Table 13 isolates each component’s contribution under identical settings (k=7, stratified 10-fold CV). The RF-weighted distance, combined with IDW voting, is the single most impactful pairing (+0.074 AUC for Full over MI + Euclidean + Majority, a row that swaps *both* the distance and the vote); isolating the distance alone, with IDW voting held fixed (Table 12), RF-weighted Euclidean still yields +0.076 AUC over plain Euclidean, confirming the distance as the dominant single factor. MI selection adds a further +0.027 (Full vs. No MI). The cumulative contribution of MI selection and the RF-weighted distance is +0.102 AUC over the minimal-component baseline (No MI + Euclidean + Majority). IDW voting is retained over majority voting because it produces the continuous probability estimates required by Revise (Equation (3)).

### 4.12. Class-Imbalance Ablation

Both cohorts are imbalanced toward the disease class (GaitPDB 66 PD/49 control; GaitNDD 39 NDD/16 control), a setting widely studied in machine learning [56]. We evaluated four balancing strategies against the unbalanced configuration—class-weighted inverse-distance voting, random oversampling, SMOTE [57], and random undersampling—applied inside each training fold (Table 14). No balancing strategy meaningfully improved AUC on either cohort: on GaitPDB only class-weighted IDW edged out the unbalanced configuration, and then only within numerical tolerance (0.862 vs. the unbalanced 0.861), while every other strategy fell below it; on GaitNDD, the unbalanced configuration retained the highest AUC (0.902). Critically, every strategy reduced sensitivity—the screening-relevant metric—while raising specificity (sensitivity fell from 0.745 to 0.626–0.700 on GaitPDB and from 0.897 to 0.617–0.792 on GaitNDD), with the GaitNDD class-weighting reduction reaching significance (p=0.008). This is expected: because the disease class is the majority in both cohorts, balancing shifts the decision boundary toward the minority control class, trading disease sensitivity for specificity. Since a screening tool prioritizes minimizing missed diagnoses, we report the unbalanced configuration and route low-confidence cases to specialist review (Revise) rather than rebalancing.

## 5. Discussion

The novelty of CogCBR lies not in any single CBR operator: weighted Euclidean distance, IDW voting, entropy-based confidence, and competence-based maintenance are all established techniques drawn from the CBR literature of the last three decades. The contribution lies in their principled *integration* into a complete R^4^ pipeline for a clinical screening domain in which prior CBR work has typically stopped at Retrieve and Reuse [20,22], combined with an explicit case-base augmentation policy that closes the loop between confident predictions, expert review, and case storage. Revise enables the system to flag uncertain or potentially unreliable predictions for specialist review; Retain provides a designed mechanism for long-term case-base quality (its longitudinal effect remains to be empirically validated); and the explanation output supports clinician verification.

Framing CogCBR through Richter’s containers [23] makes a design choice explicit: in domains where adaptation knowledge is hard to elicit, such as clinical gait screening from sensor data, it is rational to invest more in the vocabulary, similarity, and case-base containers. The empirical results agree: the strongest single contributor is similarity (the RF-weighted distance, +0.076 AUC in isolation), followed by vocabulary (MI selection, +0.027 AUC). With these containers strengthened, lightweight inverse-distance voting in the adaptation container suffices.

The Revise stage provides self-assessment of prediction reliability: at threshold ≥0.1, the pooled AUC improves from 0.868 to 0.907 at 68.7% coverage. Subject #109 (Section 4.6) illustrates the safety value: despite a wrong PD prediction, the low confidence (0.53) would, under a stricter triage threshold (≥0.6; Table 6), trigger specialist referral rather than an automated decision. We do not claim that the entropy-based confidence score reported here is well-calibrated in the strict probabilistic sense; a dedicated calibration analysis (Section 4.5, Table 7, Figure 7) confirms this and shows that post hoc calibration (Platt scaling, isotonic regression, RF-probability + Platt) and split conformal prediction yield well-calibrated or validly covered estimates when a calibrated risk is required, while the entropy score is retained for triage, where separation rather than calibration is what matters.

Beyond predictive metrics, an important limitation is that the present study does not include a clinician-centered evaluation, and the intended human–AI workflow deserves to be made explicit. We envisage CogCBR operating as a first-line triage aid rather than an autonomous diagnostic system. For each incoming recording, the clinician is shown not a bare label but the case-based explanation of Section 3.7: the *k* most similar prior patients, their labels and distances, the gait features driving each match, and the direction of clinical deviation, together with the contextual metadata (age, sex, recording protocol, and, where available, Hoehn & Yahr stage) that is displayed but excluded from the distance. This lets the clinician verify a prediction against patients they can inspect, and flag demographic or protocol mismatches that a scalar score would hide. The confidence threshold τ is the operating control of this workflow: lowering τ increases automated coverage at the cost of admitting less-certain predictions, while raising it routes more borderline cases to specialist review (Table 6). As we stress in Section 3.5 and Section 4.4, τ is a deployment-time parameter that must be calibrated on a target cohort before any specific value is recommended, since the costs of a missed diagnosis and an unnecessary referral are asymmetric and site-specific. Several workflow-integration challenges remain open and are not resolved by the present cross-sectional study: eliciting confirmed labels at clinical follow-up at a realistic rate (the confirmation-rate sweep of Section 4.8 brackets this), avoiding automation bias when an explanation is persuasive but wrong (Case C in Section 4.6 is a deliberate example), integration with electronic-health-record and sensor-acquisition systems, and the regulatory requirement for prospective validation as a clinical decision-support tool. A dedicated clinician-in-the-loop usability study—measuring how retrieved cases and confidence flags affect referral decisions, reading time, and trust—is an important direction for future work.

Beyond classification performance, the computational profile reported in Section 4.10 is directly relevant to the wearable-sensor screening setting that motivates this paper. With a deployment payload of 8.4 KB and a per-subject inference latency below 0.1 ms, CogCBR fits comfortably on the embedded processors typical of force-sensitive insoles, mobile health terminals, and edge gateways. Importantly, this efficiency does not trade off against the clinically useful capabilities of the framework: case-level explanations, neighbor-retrieval evidence, and entropy-based confidence are all preserved, which is not the case for the equally compact XGBoost baseline. We therefore view edge deployability not as an isolated engineering metric but as a structural property of the CBR design that complements its interpretability for sensor-based screening.

Two statistical issues warrant explicit discussion. First, regarding multiple comparisons on GaitPDB, the per-baseline *p*-values in Table 1 are uncorrected pairwise Wilcoxon tests (summarized before and after correction per baseline in Table 5); under Bonferroni correction at α/16≈0.003 only the 1D-CNN comparison (p=0.002) survives. We therefore do *not* claim that CogCBR is significantly better than every baseline after correction. What we do claim is that (i) CogCBR’s mean per-fold AUC ranks at or above every baseline on both datasets, (ii) the direction of the effect is consistent across all 16 baselines, and (iii) the architecture provides confidence-based triage and case-based explanations that none of the baselines, including the higher-AUC ones, provide. The uncorrected per-baseline p<0.05 pattern is best regarded as suggestive evidence warranting larger-cohort confirmation. Second, regarding variance on GaitNDD, the AUC ranking does not reach statistical significance against the strongest baselines (Random Forest 0.887, Rehman-2019 0.872): with only 55 subjects the per-fold variance (±0.111) is large relative to the absolute AUC differences (∼0.015–0.030). Establishing a stronger empirical separation will require larger multi-center cohorts and a fully nested CV protocol with subtype-stratified analysis.

A third caveat concerns *baseline fairness*. Group A baselines use scikit-learn defaults, while CogCBR’s *k*, *n*, and distance metric are tuned. We deliberately fixed baseline hyperparameters to limit researcher degrees of freedom on the baseline side, but this design choice introduces a tuning asymmetry. This asymmetry is evaluated using the fully tuned-vs.-tuned comparison of Section 4.3 (Table 3 and Table 4), in which each baseline receives the same inner-CV tuning budget that CogCBR receives. Under matched tuning, CogCBR’s nested AUC (0.842) remains the highest on GaitPDB but is statistically indistinguishable from the strongest tuned baseline (GBM, 0.836; p=0.242), and on GaitNDD the ranking is not separable within the per-fold noise (tuned Random Forest leads at 0.913). The claim of this paper is therefore not that CogCBR *outperforms* all baselines in a strict sense, but rather that it *achieves competitive top-ranked performance while additionally providing confidence-based triage and case-based explanations*.

Adding the re-implemented Wahid-2015 [7] and Rehman-2019 [8] pipelines as direct baselines (Group C in Table 1) addresses the gap that these works have frequently been discussed but not directly compared. CogCBR’s mean AUC ranks above both on GaitPDB at uncorrected p<0.05 while remaining within the noise band on GaitNDD; neither comparison survives Bonferroni correction in isolation. The pattern suggests that any modest gains over published gait pipelines come primarily from the integrated similarity, selection, and triage design rather than from the underlying feature set, which is broadly comparable. Relative to deep learning baselines, CogCBR’s edge on these small cohorts (115 and 55 subjects) reflects the fact that similarity-based reasoning avoids high-dimensional parameter estimation; this should not be read as evidence that deep learning is generally weaker in this domain, since on larger multi-center cohorts the picture is likely to change. The LSTM-raw sensitivity analysis (Section 3.9) helps disentangle the cause of the deep baselines’ weakness: training the LSTM on the raw force time series rather than the engineered features leaves AUC essentially unchanged (0.781 versus tabular 0.777, Wilcoxon p=0.39), indicating that limited sample size, rather than feature representation or sequence-model architecture, is the dominant bottleneck for the deep baselines on these cohorts.

The Retain stage *is designed* to maintain case-base quality as new sensor recordings accumulate. The augmentation policy of Section 3.6, under which only label-verified cases enter the case base, is intended to prevent the self-confirmation bias that simple confidence-based augmentation would introduce. The competence analysis in Section 4.7 characterizes the static case base under 10-fold CV but does not, by itself, demonstrate that the policy improves performance over time. A deployment-style streaming simulation (Section 4.8, Table 9) validates this policy longitudinally: the label-verified policy injects zero label noise versus ≈11 erroneous labels for naive self-training, exceeds a static no-growth baseline, and matches an all-true-label no-curation reference in final AUC while bounding case-base growth. This remains a resampling-based simulation on a cross-sectional cohort; prospective longitudinal clinical validation is still required.

Several limitations of the present study should be acknowledged so that readers can calibrate the strength of the reported findings. Sample sizes (55–115 subjects) are typical for clinical gait sensor studies but limit statistical power, particularly on GaitNDD. The RF-weighted distance assumes globally consistent feature importance, which may not hold across NDD subtypes (PD, HD, ALS); subtype-aware local weighting is a natural extension. Beyond these structural limitations, we list five further methodological caveats:

1. **MI selection on the full dataset.** MI ranking is computed once on the full dataset, which is common practice for univariate filters [40] but may introduce optimistic bias relative to a fully nested protocol [58]. The nested audit of Section 4.3, which recomputes MI ranking inside each training fold, quantifies this bias directly and finds it small on GaitPDB (ΔAUC=0.019).
2. **Hyperparameter selection on the evaluation folds.** The values n=18 and k=7 were selected at the AUC peak of the same 10-fold protocol used for the main results, rather than via an inner-loop nested CV. To quantify the resulting optimism, we conducted a nested cross-validation audit (Section 4.3), in which median imputation, standardization, MI ranking, RF feature-weight estimation, and the selection of *n* and *k* are all performed strictly inside the training folds. The audit shows the optimism is small on GaitPDB (ΔAUC=0.019; nested 0.842 versus fixed-parameter 0.861) and larger but within the high-variance regime expected for the 55-subject GaitNDD cohort (nested 0.835±0.168). Baseline fairness is further evaluated in the tuned-vs.-tuned comparison (Table 3 and Table 4), in which CogCBR remains the top performer on GaitPDB—not significantly different from tuned GBM (p=0.242)—and competitive within the noise band on GaitNDD.
3. **Retain validated by simulation, not yet prospectively.** The static competence analysis (Section 4.7) is complemented by a deployment-style streaming simulation (Section 4.8) in which the case base grows over time under a range of clinical-feedback rates. The simulation confirms that the label-verified augmentation policy avoids the label-noise accumulation of naive self-training (0 versus ≈11 injected errors), exceeds a static no-growth baseline, and matches an all-true-label no-curation reference in final AUC while bounding case-base growth. This remains a resampling-based simulation on a cross-sectional cohort; prospective longitudinal clinical validation is still required.
4. **Dataset-specific neighborhood choice on GaitNDD.** The GaitNDD analysis uses k=3 rather than k=7 to avoid an excessive neighborhood-to-minority-class ratio under the smaller cohort and reduced feature space (Section 4.9); a fully nested protocol on a larger external cohort is required to confirm this choice without dataset-specific tuning.
5. **Cohort composition.** GaitPDB subjects are diagnosed PD patients (Hoehn & Yahr 2–3) rather than pre-clinical or at-risk subjects, and GaitNDD provides only stride-interval data rather than the full insole pipeline. The present study therefore evaluates gait-based screening support and classification of established NDD-related gait patterns; demonstrating utility for true pre-symptomatic detection would require dedicated pre-clinical cohorts with longitudinal follow-up.

The present study is therefore best viewed as a methodological validation based on available cross-sectional datasets, rather than a prospective clinical deployment study. Although the cross-validation, independent-cohort evaluation, and streaming simulation support the robustness of CogCBR, the Retain stage and triage threshold still require prospective, clinician-in-the-loop validation before clinical use.

The augmentation policy assumes that specialist labels are available for low-confidence cases; in deployment, this requires integration with the clinical workflow and, at the regulatory level, prospective validation as a clinical decision support tool. Future work should explore multi-center sensor validation, subtype-aware local weighting, prospective clinical evaluation of the triage workflow, a full multi-cohort calibration study extending the single-cohort calibration analysis of Section 4.5, a clinician-in-the-loop usability evaluation of the explanation-and-triage workflow described in Section 5, and prospective longitudinal clinical evaluation of the Retain stage extending the deployment-style streaming simulation of Section 4.8.

## 6. Conclusions

In this paper, we have presented **CogCBR**, a clinical CBR framework that implements the complete R^4^ cycle for wearable-sensor-based gait screening support in NDDs. Framing the design through Richter’s four knowledge containers makes explicit the trade-off CogCBR exploits: invest in vocabulary, similarity, and case-base curation rather than in complex adaptation knowledge. CogCBR achieves AUC = 0.861 on PhysioNet GaitPDB and AUC = 0.902 on an independent-cohort reproducibility evaluation on GaitNDD, with competitive performance on both datasets—statistically on par with the strongest tuned baselines on GaitPDB under a matched-tuning comparison. Beyond ranking, it delivers confidence-based clinical triage (Revise), a competence-based case-base maintenance policy under an explicit label-verified augmentation rule (Retain, whose longitudinal behavior we validate in a deployment-style streaming simulation), and intrinsic case-based explanations—capabilities that none of the classical baselines provide. The computational profile (0.080 ms per-sample inference, 8.4 KB deployment payload) supports CogCBR’s suitability for wearable-sensor and edge-health screening scenarios.

We explicitly do *not* claim conclusive multi-comparison-corrected superiority over every baseline (only the 1D-CNN comparison survives Bonferroni correction; Table 5) or utility for pre-symptomatic detection (which would require dedicated pre-clinical cohorts). The longitudinal augmentation policy is validated in a deployment-style streaming simulation (Section 4.8) but not yet in a prospective clinical study, and a calibrated triage operating point is characterized on a single cohort (Section 4.5) but still requires multi-cohort calibration before a specific threshold can be recommended for clinical use. Under a stricter cross-modality source-to-target transfer (train on GaitPDB, test on GaitNDD), CogCBR does not exceed the strongest classical baseline, and this limitation is reported in the source-to-target transfer experiment. These remain directions for future work, alongside multi-center and prospective clinical evaluation of the sensor-driven pipeline, transfer- and meta-learning techniques to shorten the cold-start phase in new clinical settings, and evaluation on richer wearable modalities such as inertial measurement units and combined IMU + insole recordings.

## Figures and Tables

**Table 1:** Screening performance under stratified 10-fold CV on the primary GaitPDB cohort and an independent-cohort reproducibility evaluation on GaitNDD (within-cohort 10-fold CV). Best in **bold**. † = deep learning; ‡ = domain-specific gait baseline (re-implemented). ^∗^ *p* < 0.05, ^∗∗^ *p* < 0.01 vs. CogCBR (one-sided Wilcoxon signed-rank, *uncorrected for multiple comparisons*). The Bonferroni-corrected significance at α/16≈0.003 is reached only by the 1D-CNN comparison (p=0.002); see Section 5. “—” = not evaluated; baselines requiring time-series input or that failed to converge on n=55 were omitted on GaitNDD (Section 3.9). LSTM-raw is reported as a supplementary raw-sequence configuration and is *not* counted among the 16 baselines used for multiple-comparison correction. CogCBR achieves sensitivity = 0.745 on GaitPDB and 0.897 on GaitNDD; per-baseline sensitivities are omitted from the table for compactness and may not follow the same ranking as accuracy.

Method	GaitPDB (115 subj.)					GaitNDD (55 subj.)	
	AUC	Acc	F1	Spec	*p*	AUC	*p*
CogCBR (Ours)	0.861	0.800	0.801	0.880	—	0.902	—
Domain-specific gait baselines (Group C)							
Wahid-2015 ‡ [7]	0.798	0.722	0.760	0.640	0.031 ^∗^	0.804	0.203
Rehman-2019 ‡ [8]	0.821	0.748	0.788	0.660	0.049 ^∗^	0.872	0.438
Generic ML baselines (Group A)							
XGBoost	0.809	0.730	0.772	0.630	0.049 ^∗^	0.812	0.219
Random Forest	0.793	0.738	0.776	0.645	0.049 ^∗^	0.887	0.594
Log. Regr.	0.799	0.749	0.781	0.695	0.037 ^∗^	0.833	0.406
SVM (Linear)	0.793	0.713	0.762	0.595	0.040 ^∗^	—	—
SVM (RBF)	0.769	0.694	0.748	0.545	0.063	0.821	0.266
*k*-NN (k=5)	0.763	0.720	0.753	0.665	0.014 ^∗^	0.833	0.031 ^∗^
Naive Bayes	0.783	0.694	0.682	0.790	0.055	0.858	0.406
Decision Tree	0.714	0.724	0.763	0.655	0.010 ^∗∗^	0.783	0.055
MLP	0.781	0.739	0.766	0.690	0.013 ^∗^	—	—
GBM	0.836	0.740	0.785	0.610	0.121	0.850	0.453
Deep learning baselines (Group B)							
Deep MLP †	0.754	0.686	0.715	0.650	0.012 ^∗^	—	—
1D-CNN †	0.722	0.630	0.622	0.690	0.002 ^∗^	—	—
LSTM †	0.777	0.693	0.726	0.650	0.020 ^∗^	—	—
TabNet †	0.706	0.706	0.724	0.715	0.021 ^∗^	—	—
Supplementary raw-sequence baseline (not in 16-baseline correction)							
LSTM-raw †	0.781	0.703	0.731	0.660	0.023 ^∗^	—	—

**Table 2:** Nested cross-validation bias audit. Fixed-parameter rows reproduce the protocol used for the main comparison (Table 1); nested rows recompute median imputation, standardization, MI ranking, RF feature weights, and the selection of *n* and *k* strictly inside each training fold. Nested values are mean ± SD over the 10 outer folds.

Dataset	Protocol	AUC	Acc	F1	Sens.	Spec.
GaitPDB	Fixed-parameter	0.861	0.800	0.801	0.745	0.880
	Nested (ΔAUC=−0.019)	0.842±0.093	0.758±0.123	0.781±0.123	0.781±0.186	0.735±0.155
GaitNDD	Fixed-parameter	0.902	—	—	—	—
	Nested (ΔAUC=−0.067)	0.835±0.168	0.857±0.104	0.893±0.081	0.892±0.135	0.750±0.335

**Table 3:** Tuned-vs.-tuned comparison on GaitPDB (stratified 10-fold, seed 42; each Group A baseline tuned by inner-CV, CogCBR uses its nested AUC). *p* is the one-sided Wilcoxon signed-rank ($H_{1}$: CogCBR > baseline), uncorrected; *no* comparison survives Bonferroni correction (α/16≈0.003). CogCBR posts the highest AUC but its margin over the strongest tuned baseline (GBM) is not significant.

Method	Default AUC	Tuned AUC	*p* vs. CogCBR
CogCBR (nested)	0.861	**0.842**	—
GBM	0.836	0.836	0.242
SVM-Linear	0.793	0.823	0.216
SVM-RBF	0.769	0.798	0.097
XGBoost	0.809	0.794	0.037
Logistic Regression	0.799	0.785	0.156
Random Forest	0.793	0.784	0.006
Naive Bayes	0.783	0.783	0.043
MLP	0.781	0.748	0.005
*k*-NN	0.763	0.731	0.005
Decision Tree	0.714	0.721	0.019

**Table 4:** Tuned-vs.-tuned comparison on GaitNDD (Group A baselines that run on stride-interval data and converge; MLP and SVM-Linear are excluded as in Table 1). Per-fold AUC variance is large (SD up to ±0.24 on 55 subjects), so the ranking is not statistically separable; no comparison survives Bonferroni correction.

Method	Default AUC	Tuned AUC	*p* vs. CogCBR
Random Forest	0.887	**0.913**	0.892
*k*-NN	0.833	0.883	0.857
Naive Bayes	0.858	0.858	0.642
XGBoost	0.812	0.838	0.554
CogCBR (nested)	0.902	0.835	—
SVM-RBF	0.821	0.821	0.446
GBM	0.850	0.819	0.500
Logistic Regression	0.833	0.817	0.358
Decision Tree	0.783	0.758	0.171

**Table 5:** Multiple-comparison summary on GaitPDB. For each baseline, the uncorrected one-sided Wilcoxon signed-rank *p*-value ($H_{1}$: CogCBR > baseline, from Table 1) and whether it remains significant at the uncorrected level (α=0.05) and at the Bonferroni-corrected level (α/16≈0.003). Thirteen of sixteen comparisons are significant before correction; only the 1D-CNN comparison survives Bonferroni correction.

Baseline	*p* (uncorr.)	p<0.05	p<0.003
Wahid-2015	0.031	Yes	No
Rehman-2019	0.049	Yes	No
XGBoost	0.049	Yes	No
Random Forest	0.049	Yes	No
Logistic Regression	0.037	Yes	No
SVM (Linear)	0.040	Yes	No
SVM (RBF)	0.063	No	No
*k*-NN (k=5)	0.014	Yes	No
Naive Bayes	0.055	No	No
Decision Tree	0.010	Yes	No
MLP	0.013	Yes	No
GBM	0.121	No	No
Deep MLP	0.012	Yes	No
1D-CNN	0.002	Yes	Yes
LSTM	0.020	Yes	No
TabNet	0.021	Yes	No

Significant before correction: 13/16; survive Bonferroni: 1/16 (1D-CNN).

**Table 6:** Revise-stage confidence threshold analysis for clinical triage. AUC is computed on predictions pooled across all folds. The high-threshold rows correspond to small absolute sample sizes (∼40 subjects at ≥0.3, ∼16 subjects at ≥0.6); the accuracies on these rows describe the easiest-to-classify subset of the cohort and should not be over-interpreted as deployment estimates. The Subjects column gives the absolute number of GaitPDB subjects retained at each threshold (pooled across folds, out of 115).

Confidence ≥	Coverage (%)	Subjects (*n*)	Accuracy (%)	AUC
0.0 (no filter)	100.0	115	80.0	0.868
0.1	68.7	79	87.3	0.907
0.3	34.8	40	92.5	0.924
0.6	13.9	16	100.0	1.000

**Table 7:** Calibration comparison on GaitPDB (stratified 10-fold CV, seed 42). Calibrators and the conformal calibration set are fit within each training fold (25% held out per fold); none see the test fold (the reduced case base accounts for the slightly lower accuracy than Table 1). ECE uses 10 bins. The entropy confidence is the triage score of Section 3.5, reported here read as a probability of correctness; its high ECE reflects that role rather than a calibrated probability. Lowest ECE in **bold**.

Method	Accuracy	ECE	Brier	Conformal Cov.
Entropy confidence (current Revise)	0.764	0.491	0.466	—
Raw IDW probability	0.764	0.098	0.183	—
Platt scaling (IDW *p*)	0.661	0.038	0.202	—
Isotonic regression (IDW *p*)	0.721	0.088	0.197	—
Split conformal (CogCBR score)	0.770	0.108	0.186	0.933 (target 0.900)
RF probability + Platt	0.715	**0.035**	0.180	—

**Table 8:** Case competence distribution under the operational Retain thresholds (Section 3.6).

Operational Group	Cases	Percentage
≥0.7 (confirmed retain)	79	68.7%
0.3–0.7 (monitoring)	27	23.5%
<0.3 (flagged for specialist review)	9	7.8%

**Table 9:** Retain longitudinal simulation on GaitPDB (τ=0.1, 10 seeds, means). A = label-verified augmentation policy; B = naive self-training (predicted labels); B2 = verification ablation (A’s data flow, unverified confident labels); C = all-true-label reference with no curation (fixed at 86 cases); D = static no-growth (34 cases, held-out AUC 0.750). *p* is the paired area-under-the-learning-curve test for A vs. B. Injected label noise: A =0 throughout, B =11.0, B2 rising 2.5→9.7 with the confirmation rate. A beats B and the static baseline D at every rate and approaches C, matching it within the per-fold noise at confirmation rates ≥0.7. Because C performs no competence-based curation, A—which does—can match or marginally exceed it (0.838 vs. 0.829 at the full confirmation rate); C is therefore a no-curation reference, not a strict upper bound. A attains this while bounding case-base growth (60.8–80.9 vs. 86 cases for C).

Confirm_Rate	A	B	B2	C	D	A Size	A-vs-B *p*
0.3	0.801	0.719	0.802	0.829	0.750	60.8	0.049
0.5	0.807	0.719	0.792	0.829	0.750	66.7	0.006
0.7	0.824	0.719	0.784	0.829	0.750	72.5	0.004
1.0	0.838	0.719	0.774	0.829	0.750	80.9	0.002

**Table 10:** Source-to-target transfer: train on GaitPDB and test on GaitNDD using only shared stride-based features. Best per column in **bold**. This experiment is reported for transparency; the main claims of the paper rely on within-dataset cross-validation and the independent-cohort reproducibility evaluation on GaitNDD rather than on direct cross-modality transfer, in which CogCBR does not exceed the strongest classical baseline.

Method	AUC	Acc	F1	Sens.	Spec.
Logistic Regression	**0.832**	**0.764**	**0.817**	**0.744**	0.813
GBM	0.798	0.582	0.610	0.462	0.875
XGBoost	0.763	0.618	0.656	0.513	0.875
Random Forest	0.757	0.618	0.644	0.487	**0.938**
SVM (RBF)	0.752	0.473	0.431	0.282	**0.938**
*k*-NN	0.752	0.618	0.656	0.513	0.875
CogCBR	0.737	0.564	0.600	0.462	0.813

**Table 11:** Computational cost and deployment footprint on the GaitPDB analysis set (115 subjects). Training time is reported as wall-clock fitting time. CogCBR includes RF-based feature-weight estimation; at this scale, MI ranking and standardization introduce negligible overhead. Inference latency is measured for a single query sample (per-subject deployment scenario) and averaged over 2000 runs. Best in **bold**.

Method	Train Time (s)	Inference (ms/Sample)	Deployment Size (KB)
**CogCBR**	<0.1	**0.080**	**8.4**
Random Forest	<0.1	2.678	48.1
XGBoost	<0.1	0.510	15.0
1D-CNN	1.515	0.426	35.0
LSTM	0.980	0.369	211.7

**Table 12:** Distance metric ablation (MI-18, k=7).

Distance Metric	AUC	Accuracy	Sensitivity	Specificity
RF-weighted Euclidean	0.861	0.800	0.745	0.880
Cosine	0.813	0.748	0.790	0.685
Manhattan	0.805	0.715	0.700	0.740
Euclidean	0.785	0.764	0.729	0.815
NCA	0.766	0.688	0.686	0.685

**Table 13:** Component ablation. MI = MI feature selection (18 features); W-Euclid = RF-weighted Euclidean distance; IDW = inverse-distance weighted voting.

Configuration	AUC	Δ
Full CogCBR (MI + W-Euclid + IDW)	0.861	—
No MI (41 feat. + W-Euclid + IDW)	0.834	−0.027
MI + Euclidean + Majority	0.787	−0.074
MI + NCA + IDW	0.766	−0.095
No MI + Euclidean + Majority	0.759	−0.102

**Table 14:** Class-imbalance ablation on GaitPDB and GaitNDD (stratified 10-fold CV, seed 42; balancing applied inside each training fold). Values are means over the 10 folds. Strategy (a) is the unbalanced CogCBR configuration and reproduces the primary result (Table 1); minor differences in threshold-dependent metrics reflect numerical tolerance across re-runs. No strategy improves AUC; all reduce sensitivity and raise specificity. GaitNDD per-class metrics carry wide variance (standard deviation up to ±0.33 for specificity, owing to its 16 controls) and should be read with caution; the sensitivity reduction under class-weighting on GaitNDD is significant (0.897→0.617, p=0.008).

Strategy	AUC	Acc	F1	Sens.	Spec.
GaitPDB					
(a) None (unbalanced)	0.861	0.800	0.801	0.745	0.880
(b) Class-weighted IDW	0.862	0.748	0.736	0.657	0.880
(c) Random oversampling	0.846	0.783	0.778	0.700	0.900
(d) SMOTE	0.838	0.740	0.728	0.626	0.900
(e) Random undersampling	0.847	0.749	0.743	0.657	0.880
GaitNDD					
(a) None (unbalanced)	0.902	0.837	0.882	0.897	0.688
(b) Class-weighted IDW	0.902	0.710	0.745	0.617	0.950
(c) Random oversampling	0.858	0.750	0.774	0.692	0.900
(d) SMOTE	0.840	0.763	0.797	0.742	0.800
(e) Random undersampling	0.840	0.797	0.842	0.792	0.800

## Data Availability

The GaitPDB and GaitNDD datasets analyzed in this study are publicly available from PhysioNet (https://physionet.org). All algorithmic details required to reproduce the CogCBR pipeline are described in Section 3 and summarized in Algorithm 1; additional implementation details are available from the corresponding author upon reasonable request.

## Abbreviations

The following abbreviations are used in this manuscript:

ALS	Amyotrophic Lateral Sclerosis
AUC	Area Under the (ROC) Curve
CBR	Case-Based Reasoning
CV	Coefficient of Variation
ECE	Expected Calibration Error
GBM	Gradient Boosting Machine
GRF	Ground Reaction Force
HD	Huntington’s Disease
IDW	Inverse-Distance Weighted
IMU	Inertial Measurement Unit
*k*-NN	*k*-Nearest Neighbors
LIME	Local Interpretable Model-agnostic Explanations
LSTM	Long Short-Term Memory
MI	Mutual Information
MLP	Multilayer Perceptron
NDD	Neurodegenerative Disease
NCA	Neighborhood Components Analysis
PD	Parkinson’s Disease
RF	Random Forest
ROC	Receiver Operating Characteristic
SHAP	SHapley Additive exPlanations
SMOTE	Synthetic Minority Over-sampling Technique
SVM	Support Vector Machine
